# Fabrication of two dual-functionalized covalent organic polymers through heterostructural mixed linkers and their use as cationic dye adsorbents†

**DOI:** 10.1039/c8ra01968a

**Published:** 2018-05-23

**Authors:** Jun Dong, Feifan Xu, Zhaojun Dong, Yongsheng Zhao, Yan Yan, Hua Jin, Yangxue Li

**Affiliations:** Key Lab of Groundwater Resources and Environment, Ministry of Education, Jilin University 2519 Jiefangda Road Changchun 130021 P. R. China yangxueli@jlu.edu.cn; State Key Laboratory of Inorganic Synthesis and Preparative Chemistry, College of Chemistry, Jilin University Changchun 130012 P. R. China

## Abstract

With the rapid development of industrialization, population growth and long-term droughts, the scarcity of clean water and increasing environmental pollution are becoming critical issues. Porous organic polymer materials have been proposed as good candidates for removing pollutant compounds from water supplies. However, because of their finite synthetic chemical reactions and monotonous building blocks, fabricating covalent organic polymer materials with multiple linkages and various different multi-functional components remains a huge challenge. Herein, two dual-functionalized covalent organic polymers (JLUE-COP-1 and JLUE-COP-2) were prepared through heterostructural mixed linkers *via* imine and hydrazone linkages based on Schiff base condensation. Possessing the advantages of porosity and π-conjugated phenyl rings, as well as functional –CO–NH– and –SO_3_H building units, the resultant dual-functionalized JLUE-COPs exhibited high capabilities for the removal of the cationic dye methylene blue (MB). Experiments were carried out to investigate the effects of pH, dye concentration, contact time and temperature on the performance of the JLUE-COPs. The kinetics, equilibrium properties, thermodynamics and mechanisms were studied. It was found that the adsorption processes of the two JLUE-COPs both followed the second-order kinetic model and can be described well by the Langmuir model. Notably, the differences in the surface and pore volumes of the two JLUE-COPs lead to differences in the adsorption rate, adsorption efficiency and maximum adsorption capacity. In addition, activation energy values and some thermodynamic parameters such as Δ*G*^*Θ*^, Δ*H*^*Θ*^ and Δ*S*^*Θ*^ suggest that the adsorption processes of dyes onto the JLUE-COPs are entropy-driven, endothermic and spontaneous. Our work thus paves the way for developing functionalized COPs as a new type of platform for removing cationic dyes from wastewater.

## Abstract 2

Two dual-functionalized covalent organic polymers are synthesized from heterostructural mixed linkers by Schiff base condensation. Due partly to their porosity and π-conjugated phenyl rings these remove organic pollutants from waste water effectively.

## Graphical abstract

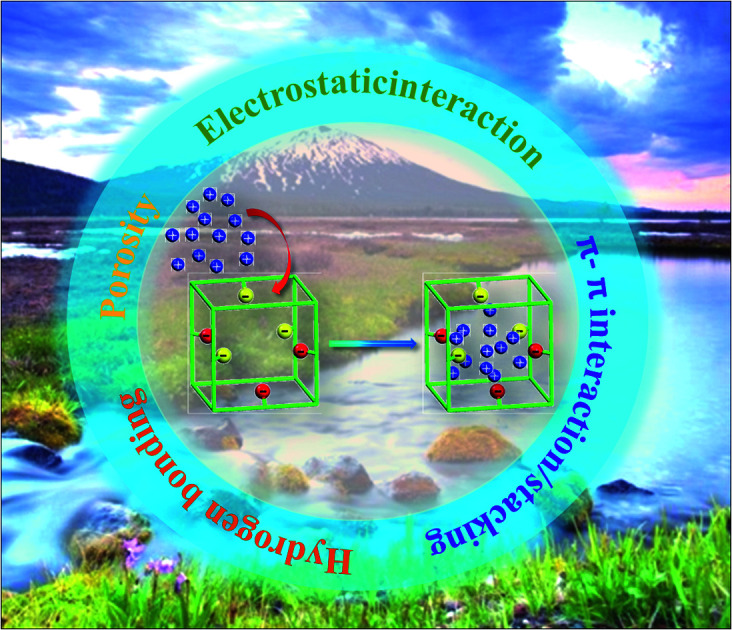

## Introduction

Water resources, whose availability significantly affects the economic and social development of all countries in the world, are seriously polluted every day. Printing ink and dye pollution in wastewater has come to the foreground as one of the main drivers behind the water resource crisis.^1^ As organic dyes are widely used in the textile, dyeing, paper and pulp, ink, petroleum refining, food processing, fertilizer and tanning industries, more than 100 000 dyes have been generated for commercial use, and 10–15% of them are usually discharged into aquatic systems.^2–5^ Organic dyes, which often exhibit undesirable colors, tastes and odors, can be harmful and toxic to human beings. Methylene blue (MB), for example, as one of the most commonly used cationic dyes, not only can cause permanent injury to the eyes of humans and animals, but can also give rise to shortness of breath upon inhalation, while producing nausea, vomiting, profuse sweating, mental confusion, painful micturition and methemoglobinemia when ingested.^6^ Beyond that, organic dyes can also absorb and reflect sunlight into the water, interfering with the growth of bacteria and affecting the photosynthesis of aquatic organisms.^7,8^ Moreover, most organic dyes are difficult to degrade, even when treated with heating and oxidation, because of their aromatic components, including benzene, naphthalene and anthracene. It is evident, therefore, that taking a firm hand to control dye pollution is becoming increasingly urgent.

So far, traditional methods for removing organic dyes from wastewaters include ion-exchange, chemical precipitation, oxidation reduction, insoluble complexing, membrane separation, evaporation recovery and electrolysis.^9^ Adsorption, which was proposed by Kagser in 1881, stands out to be superior to the other techniques in view of its comparatively low cost, high efficiency, wide application, simple design, easy operation and few harmful secondary products for the treatment of dye-contaminated wastewater. The specific properties of adsorbents have a great influence on their adsorption capacity. (1) In general, the greater the specific surface area of an adsorbent, the stronger the adsorption capacity. (2) The pore size and distribution of adsorbents also have a certain influence on the adsorption capacity and adsorption selectivity. Because of the capillary condensation and micropore filling effect, mesopores and micropores usually have a strong adsorption capacity; while the more concentrated the pore size distribution, the better the adsorption selectivity. (3) The surface chemical properties of an adsorbent are also a very important factor. According to the theory of “like dissolves like”, a polar adsorbent can easily adsorb polar substances from water; whereas, non-polar adsorbents can easily adsorb non-polar substances from water. Surface functional groups, such as oxygen-containing groups, strong base groups and weak base groups, may lead to the formation of hydrogen bonds and electrostatic interactions, so as to strengthen the adsorption capacity. Various types of sorbent materials have been applied for the adsorptive removal of toxic dyes from wastewater, including activated carbon, coal fly ash, zeolites, industrial by-products, agricultural waste, bio-adsorbents, graphene oxide and metal–organic frameworks (MOFs).^10,11^ However, there are still some problems to be solved due to the weaknesses associated with existing adsorbents, such as low yield, small surface area and low adsorbent capacity. Hence, it is of great significance to develop new types of materials for the highly effective removal of dye from wastewater.

As one type of porous organic polymers (POPs), covalent organic polymers (COPs), formed by covalent linkages with two- or three-dimensional porous structures, are of great interest due to their characteristic advantages: abundant porosity, adjustable function, diverse structure, simple preparation and low density.^12–21^ COPs have shown their great charm and distinction in wide-ranging applications, including molecular separations, fabrication of solar collectors and photodevices, heterogeneous catalysis, environmental remediation and health-related applications.^22–26^ Despite the great progress achieved in the past, attempts to increase the flexibility of the architecture and expand the functionality of COPs have been relatively unsuccessful due to limitations associated with the syntheses and the monotonicity of the building blocks.^27,28^ Moreover, the properties of a functional porous framework are crucially determined by the characteristics of the building units.

Very recently, our group developed a three-component pha-HcOP-1 to serve as a host framework for iodine enrichment.^29^ In this contribution, we adopted the heterostructural mixed linker approach to achieve a rigid and flexible combination, successfully synthesizing two dual-functionalized COPs named JLUE-COPs with multiple linkages in high yield. The three-component JLUE-COPs were constructed using benzene-1,3,5-tricarbohydrazide (BTCH), terephthalaldehyde (TPA) or isophthalaldehyde (IPA) and 2,5-diaminobenzenesulfonic acid (DABA) *via* a Schiff base reaction in DMSO as a solvent. The introduction of –CO–NH– groups and strongly acidic anionic –SO_3_H groups endows the JLUE-COPs with negative charge and highly hydrophilic features, allowing the materials to be used to remove organic pollutants from wastewater effectively. The adsorption mechanisms are mainly related to the abundant porosity, electrostatic interaction, π–π electron donor–acceptor (EDA) interaction and hydrogen bonding between JLUE-COPs and MB.^30,31^ Herein, JLUE-COPs were studied as a new type of highly-efficient cationic dye adsorbents in aqueous solution. Moreover, to the best of our knowledge, there are few reports on the use of three-component COPs for the removal of cationic dyes.

## Experimental section

### Reagents and materials

Unless otherwise noted, all starting reagents were obtained from commercial suppliers and used without further purification. Trimethyl benzene-1,3,5-tricarboxylate and benzene-1,3,5-tricarbohydrazide were synthesized according to previous reports.^32,33^

### Apparatus

Thermogravimetric analyses (TGA) were performed using a Netzch Sta 449c thermal analyzer system at the heating rate of 5 °C min^−1^ in N_2_ atmosphere. CHN elemental analyses were collected using an Elementar model Vario Micro analyzer. The Fourier transform infrared (FT-IR) spectra were recorded using a Nicolet Impact 410 Fourier transform infrared spectrometer. The N_2_ adsorption isotherms were measured on a Micromeritics ASAP 2020 surface area and porosity analyzer. The powder X-ray diffraction (PXRD) spectra were collected using a Rigaku D/MAX2550 diffractometer with Cu Kα radiation (*λ* = 1.54178 Å). Transmission electron microscopy (TEM) images and high-resolution transmission electron microscopy (HR-TEM) images were recorded on a FEI Tecnai G2F20 s-twin D573 with an acceleration voltage of 300 kV. Field-scanning electron microscopy (FE-SEM) images were obtained on a JEOLJXA-840 under an accelerating voltage of 15 kV. The solid-state ^13^C cross-polarization/magic-angle spinning nuclear magnetic resonance (CP/MAS NMR) spectra of JLUE-COPs were obtained at 5 kHz. The zeta potentials were measured at various pH values with a JS94H (Shanghai, China).

### Synthesis of JLUE-COP-1

Benzene-1,3,5-tricarbohydrazide (BTCH, 0.2 mmol, 0.05 g), terephthalaldehyde (TPA, 0.6 mmol, 0.08 g), 2,5-diaminobenzenesulfonic acid (DABA, 0.3 mmol, 0.056 g) and CF_3_COOH (TFA, 2 d) were added into a solution of dimethyl sulphoxide (DMSO, 10 mL). A dark brown precipitate (JLUE-COP-1) was afforded in 93% yield after heating at 100 °C for 1 h. Elemental analysis (wt%) calcd for {C_26_H_19_N_8_O_6_S}_*n*_: C 54.64, H 3.35, N 19.61; found: C 54.83, H 3.23, N 19.85.

### Synthesis of JLUE-COP-2

JLUE-COP-2 was also synthesized using the same method with isophthalaldehyde (IPA, 0.6 mmol, 0.08 g) as a reactant. Benzene-1,3,5-tricarbohydrazide (BTCH, 0.2 mmol, 0.05 g), isophthalaldehyde (IPA, 0.6 mmol, 0.08 g), 2,5-diaminobenzenesulfonic acid (DABA, 0.3 mmol, 0.056 g), and CF_3_COOH (TFA, 2 d) were added into a solution of dimethyl sulphoxide (DMSO, 10 mL). A dark brown precipitate (JLUE-COP-2) was afforded in 93% yield after heating at 100 °C for 1 h. Elemental analysis (wt%) calcd for {C_26_H_19_N_8_O_6_S}_*n*_: C 54.64, H 3.35, N 19.61; found: C 54.57, H 3.12, N 19.78.

### Determination of pH of zero charge

The point of zero charge (PZC) for the JLUE-COPs was determined according to the reported literature by boiling 100 mL deionized water for 20 min to eliminate the dissolved CO_2_ and quickly cooling and capping the solution.^34^ 0.2 g of JLUE-COPs was placed in 15 mL of the CO_2_-free water, and the mixture was then sealed and continuously agitated for 48 h at room temperature before measuring the solution pH, taken as the point of zero charge.

### Batch adsorption studies

Adsorption of MB by the JLUE-COPs was carried out with batch experiments. The pH of the solution was adjusted using either 0.1 mol L^−1^ HCl or 0.1 mol L^−1^ NaOH solutions. In order to obtain the adsorption isotherms of the dyes, 30 mg of JLUE-COP adsorbents were immersed in 30 mL of MB solution (100–500 mg L^−1^) and stirred on a rotating shaker at room temperature for a period of time. Then the absorbance of the supernatant was measured by using UV-vis at various time intervals at the absorbance wavelength of 667 nm for MB. Moreover, for the sake of calculating the residue amount of MB in the solution, a series of MB solutions with different concentrations were also detected to obtain a calibration plot of standard MB.

Eqn (1)–(3) were used to calculate the removal efficiency (*E*, %), the adsorption amount of dyes by JLUE-COPs at a desired time *t* (*q*_*t*_, mg g^−1^) and the dye adsorption capacity of JLUE-COPs at equilibrium (*q*_e_, mg g^−1^), respectively:^35^1
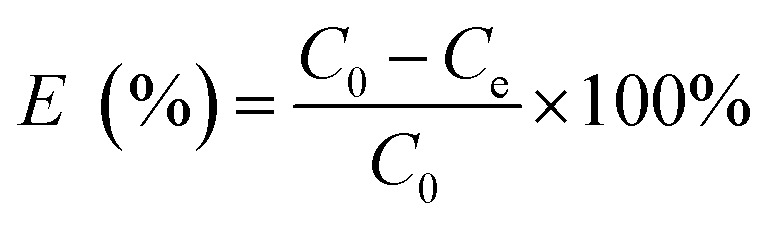
2
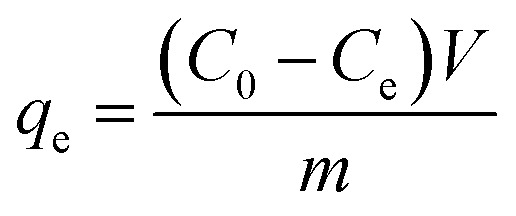
3
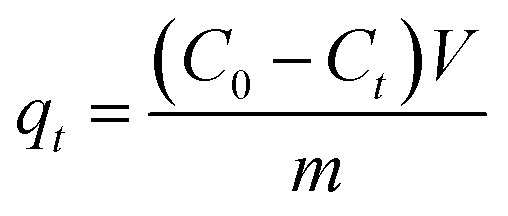
where *C*_0_ (mg L^−1^) and *C*_e_ (mg L^−1^) are the initial concentrations of dye and the equilibrium concentrations of dye, respectively; *m* (g) is the mass of adsorbent used; *V* (L) is the total volume of solution used.

## Results and discussion

### Characterization of the adsorbent

**Scheme 1:**
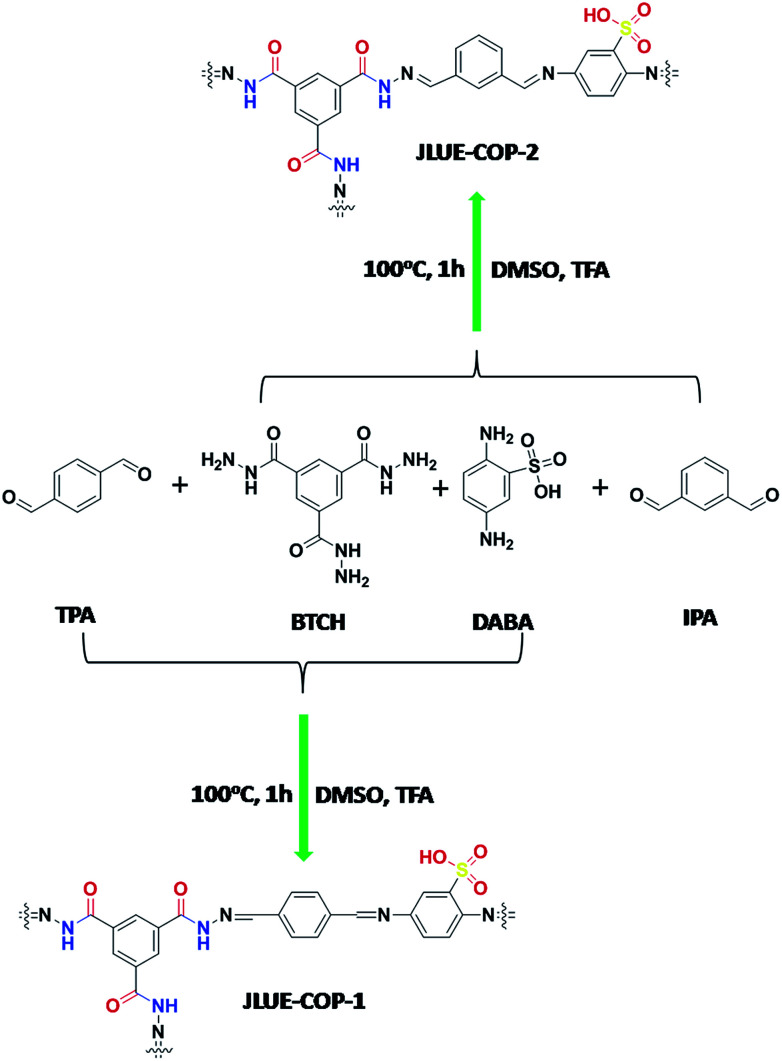
Schematic illustration of JLUE-COPs.

Typically, JLUE-COPs were synthesized using a solvothermal reaction of benzene-1,3,5-tricarbohydrazide (BTCH), terephthalaldehyde (TPA) or isophthalaldehyde (IPA) and 2,5-diaminobenzenesulfonic acid (DABA) in dimethyl sulfoxide (DMSO) with CF_3_COOH (TFA) as a catalyst, at 100 °C for 1 hour (Scheme 1). The obtained JLUE-COPs were insoluble in common organic solvents including acetone, methanol, ethanol, tetrahydrofuran, dichloromethane, chloroform and dimethyl sulfoxide.

**Fig. 1:**
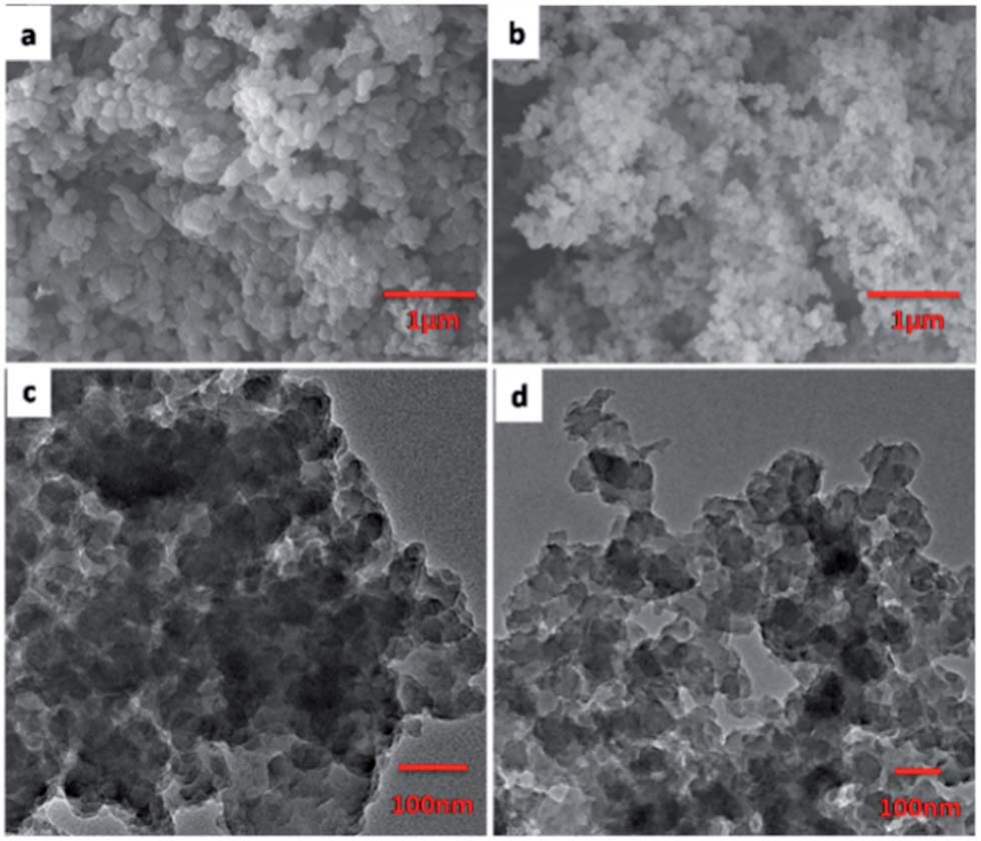
SEM images of JLUE-COP-1 (a) and JLUE-COP-2 (b); TEM images of JLUE-COP-1 (c) and JLUE-COP-2 (d).

The FE-SEM images revealed that the JLUE-COPs are composed of aggregated nanoparticles with average diameters of about 50 nm. The nanoparticles are irregular in shape without a well-defined morphology (Fig. 1a and b). To probe the long-range structure of the JLUE-COPs, powder X-ray diffraction (PXRD) and transmission electron microscopy (TEM) were performed. The TEM images (Fig. 1c and d) and HR-TEM images (Fig. S2†) indicate that the textures of the JLUE-COPs are largely amorphous. The powder X-ray diffraction patterns of the JLUE-COPs also indicate that the polymers are non-crystalline in nature (ESI, Fig. S1†). The EDS spectra of the JLUE-COPs were collected, which exhibited the expected constituents, such as S, O, H and C (Fig. S3†).

**Fig. 2:**
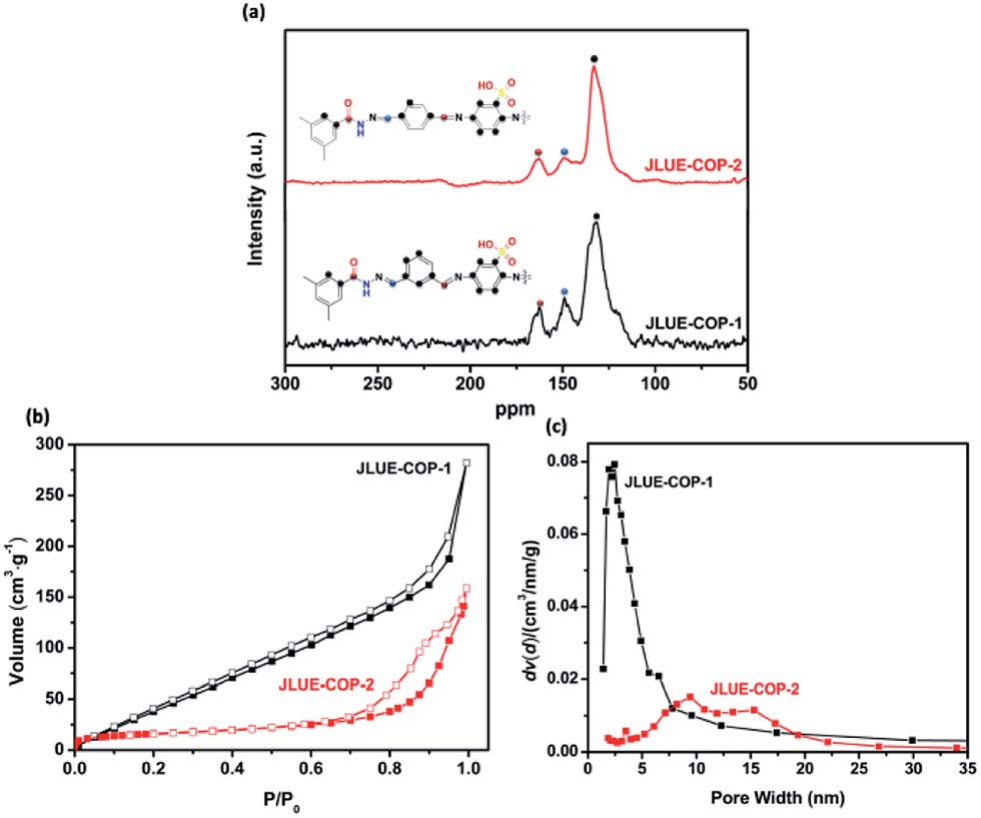
(a) ^13^C CP/MAS NMR spectra of JLUE-COPs; (b) nitrogen adsorption and desorption isotherms measured at 77.3 K; and (c) pore size distribution of JLUE-COPs.

The structural information of the JLUE-COPs was obtained using spectral methods such as ^13^C CP/MAS NMR and FT-IR. Solid-state ^13^C CP/MAS NMR analysis confirmed the successful self-assembly of imine and hydrazone bonds (Fig. 2a). For the two JLUE-COPs, the peaks at 149.6 ppm indicate the presence of hydrazine bonds (–CN–NH–), while the characteristic signals at 163.6 ppm can be assigned to both amide carbonyl bonds (–CO–NH–) and aminal bonds (C=N), respectively. Moreover, the other signals at around 120 ppm are due to aromatic carbons.^36^ Furthermore, the FT-IR spectra of JLUE-COPs (Fig. S4†) showed that the stretching modes at 1249 cm^−1^ correspond to the characteristics of *ν*_C=N_ moieties. The characteristic peaks at 1030 cm^−1^, 1074 cm^−1^ and 1494 cm^−1^ in the JLUE-COPs can be assigned to the O=S=O symmetric and asymmetric stretching bands, which indicate the existence of sulfonic acid groups. These results are in agreement with previous reports.^37^

As shown in Fig. S6,† the two JLUE-COPs show a similar thermal stability as investigated by TGA under a nitrogen atmosphere. According to the TGA curves of the two JLUE-COPs, they both exhibited a mass loss of almost 25% before 183 °C, due to the removal of guest molecules. Then, the collapse of the framework leads to a mass loss of about 19% in the range from 325 to 393 °C. N_2_ adsorption–desorption measurements were performed at 77 K by evaluating the porous properties of the two JLUE-COPs. As shown in Fig. 2b, both of the two JLUE-COPs display type II isotherms according to the IUPAC classification. JLUE-COP-2 possesses a lower Brunauer–Emmett–Teller (BET) surface area and total pore volume (64.7 m^2^ g^−1^ and 0.24 cm^3^ g^−1^) than JLUE-COP-1 (236.7 m^2^ g^−1^ and 0.52 cm^3^ g^−1^). This could be due to the steric hindrance of the constituent monomers, which have different configurations in the flexible non-ordered organic polymers. Moreover, the pore size distribution of JLUE-COPs, which was calculated by non-local density functional theory (NLDFT),^38^ identified the coexistence of micropores and macropores (Fig. 2c).

### Adsorption mechanism

**Fig. 3:**
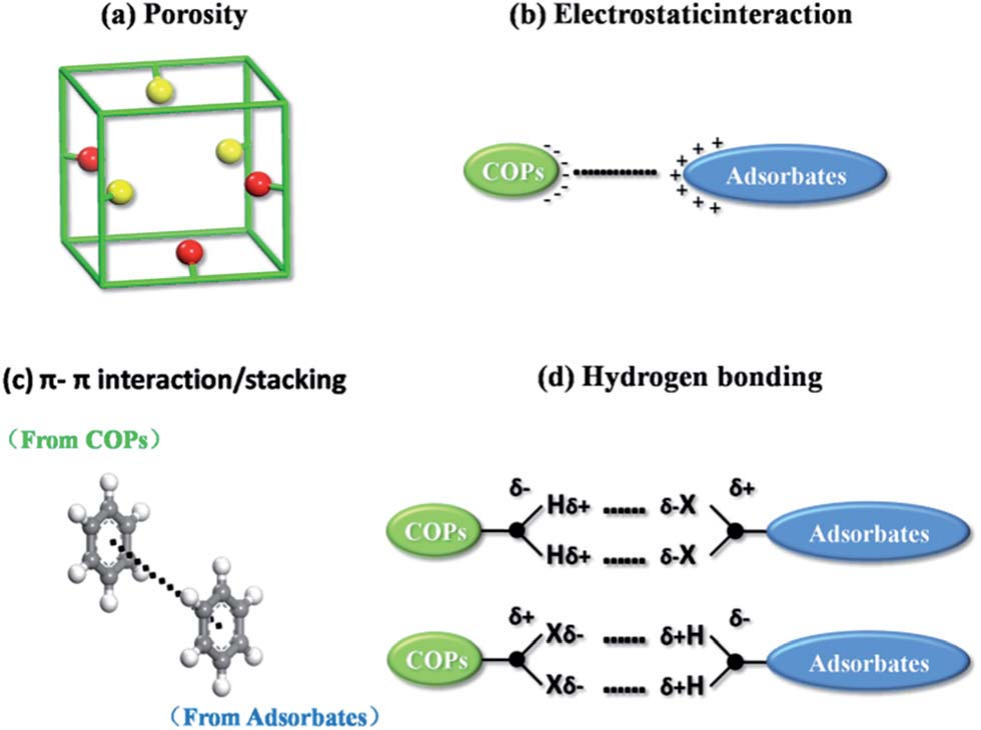
Schematic diagram of possible mechanisms for the adsorptive removal of MB.

The dye cation adsorption by JLUE-COPs was found to proceed through four main removal pathways, which take place simultaneously: abundant porosity, electrostatic interaction, π–π electron donor–acceptor (EDA) interaction and hydrogen bonding between JLUE-COPs and MB. It could be explained as the following four aspects (Fig. 3):

(a) The adsorption process mainly occurs between the adsorbate and the porous adsorbent. Therefore, the porosity of the adsorbent is very important for the adsorption process. As a consequence, the excellent specific surface area, and the simultaneous existence of micropores and macropores in JLUE-COPs provide a good breeding ground for dye adsorption.^39^

(b) The negatively charged JLUE-COPs can easily undergo interactions with positive dye cations, due to the most frequent phenomenon that occurs during the adsorption progress, *i.e.* electrostatic interactions. The net surface charge of JLUE-COPs not only depends on the pH of the solution, but is also related to the functional groups on the COPs. Herein, the JLUE-COPs exhibit charge attraction with such –CO–NH– and –SO_3_H functional groups, causing MB to flocculate. Simultaneously, adsorbed MB is synergistically removed.

(c) Another effect is believed to be the π–π electron donator–acceptor (EDA) effect, which has been treated as one of the predominant driving forces for the sorption of MB by JLUE-COPs. The –CO–NH– and –SO_3_H groups endow the JLUE-COPs with a π-electron-rich framework, and can function as electron donators. Meanwhile, the positively charged anthracene rings in MB cations can act as π-electron acceptors due to the electron deficiency.

(d) The last possible mechanism is hydrogen bonding, which has been widely proven to be involved in the sorption of polar organic pollutants. The –NH– functional groups of JLUE-COPs act as hydrogen bonding donors to form hydrogen bonds with MB, whereas the polar functional groups in MB, such as S and N, serve as hydrogen bonding acceptors. Similarly, functional groups in JLUE-COPs, such as C=O and –SO_3_H, can also act as hydrogen bonding acceptors and form hydrogen bonds with –CH_3_ in MB. Moreover, the hydrophilic functional groups of JLUE-COPs will form hydrogen bonds with water molecules, and can be treated as surface functional groups, providing large amounts of –OH for the JLUE-COPs.^40^ The bound water molecules offer more hydrogen bonding donors and acceptors and thus greatly improve the adsorption affinity of MB for the additive effect of multiple hydrogen bonds.

**Fig. 4:**
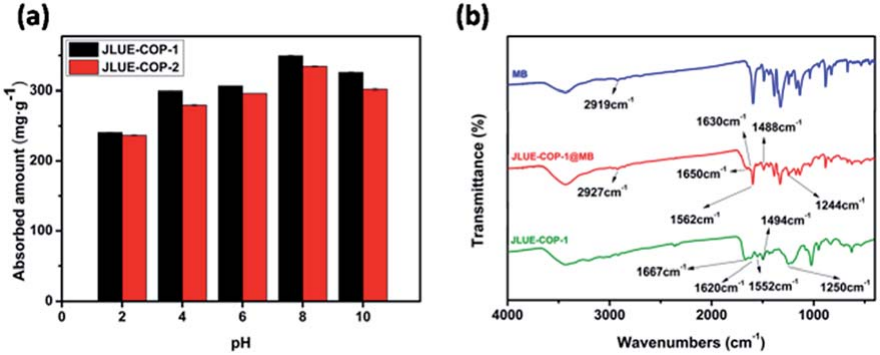
(a) Effect of solution pH on MB adsorption by JLUE-COPs. Data are presented as mean ± SD (*n* = 3); (b) FT-IR spectra of MB, JLUE-COP-1 and JLUE-COP-1@MB.

Since electrostatic interactions are regarded to play a leading role during the adsorption process, zeta potential values were obtained to get a better understanding.^41,42^ As shown in Fig. S7,† the obtained zeta potential values of JLUE-COPs decreased with increasing solution pH, and the PZC values were determined to be about 1.0 for JLUE-COP-1 and 1.3 for JLUE-COP-2, respectively. When pH < PZC, the surfaces of the JLUE-COPs were positively charged; when pH > PZC, the surfaces of JLUE-COPs were negatively charged. Based on the law of interaction between charges, the adsorption at pH < 2 would be unfavorable for dye cations because of the mutual repulsion. Therefore, the effect of the initial solution pH on the adsorption of dyes by JLUE-COPs was observed at a range of pH values from 2.0 to 10.0 (Fig. 4a). At lower pH values, protons occupy most of the adsorption sites on the adsorbent surface, leading to a poor adsorption of dye due to the competition between protons and dye molecules. In alkaline medium, both the –CO–NH– and –SO_3_H groups of JLUE-COPs are deprotonated, the surface of JLUE-COPs becomes negatively charged, and therefore, the adsorption takes place more easily. The results demonstrate that the maximal adsorption capacities for MB adsorption by JLUE-COPs reach their highest values at pH 8. Thus, an optimal pH of 8 was selected for all adsorption studies.

IR analysis was employed to go a step further and set forth the adsorption mechanism of MB by JLUE-COPs. The FT-IR spectra for JLUE-COPs, MB and JLUE-COPs@MB are shown in Fig. 4b and S5†. The C=C vibration of JLUE-COPs at 1620 cm^−1^ blue-shifted to 1630 cm^−1^ after the adsorption of MB, indicating the presence of π–π electron donor–acceptor (EDA) interactions between the phenyl rings of JLUE-COPs and MB. Both the amide peak at 1552 cm^−1^ observed for JLUE-COPs and –CH_3_ peak of MB at 2919 cm^−1^, blue-shifted toward to 1562 cm^−1^ and 2927 cm^−1^ after the adsorption of MB, respectively. Meanwhile, three bonds at 1667, 1494 and 1250 cm^−1^, assigned to the C=O and O=S=O stretching vibrations in JLUE-COPs and the C–N stretching vibration in MB, red-shifted to 1650, 1488 and 1244 cm^−1^, respectively. The results indicate multiple hydrogen bonding interactions between the –CH_3_ and C–N functional groups of MB and the –CO–NH– and –SO_3_H functional groups of JLUE-COPs.^43^

### Effect of adsorbent dose

**Fig. 5:**
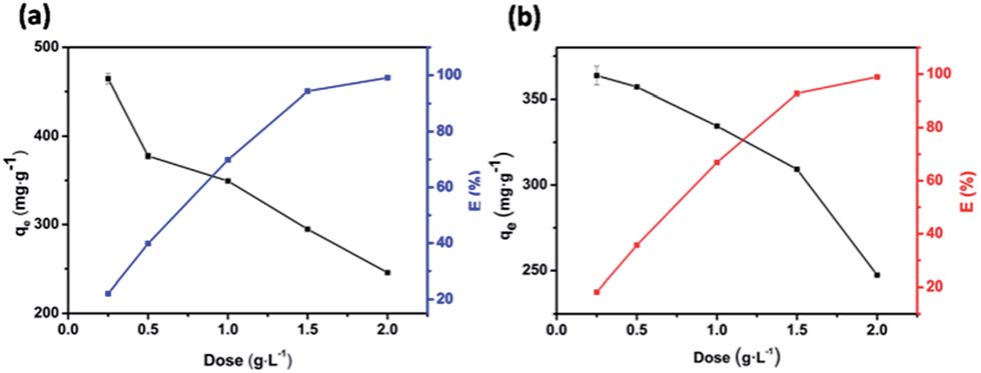
Variation in the removal efficiency *E* and adsorptive capacity *q*_e_ of JLUE-COP-1 (a) and JLUE-COP-2 (b) as a function of adsorbent dose. Data are presented as mean ± SD (*n* = 3).

The influence of adsorbent dosage on the dye removal efficiency *E* and adsorptive capacity *q*_e_ of JLUE-COPs was investigated, as shown in Fig. 5. In a batch system, various adsorbent dosages were tested to evaluate the optimal value for a fixed concentration of dyes at 500 mg L^−1^ for JLUE-COPs. In the case of JLUE-COP-1, it is worth noting that with increasing adsorbent dosage, *E* increased from 23.23% to 98.25%, while *q*_m_ decreased from 465 mg g^−1^ to 245 mg g^−1^. For JLUE-COP-2, *E* increased from 18.18% to 98.14%, while *q*_m_ decreased from 363 mg g^−1^ to 244 mg g^−1^. It is easy to understand the variations between the removal efficiency and the adsorptive capacity. As a whole, as the amount of adsorbent increases, the removal efficiency is improved due to the increased surface area and binding sites for dye adsorption. Nevertheless, the amount of adsorbate in contact with the adsorbent decreases, resulting in a decrease in the adsorptive capacity. In terms of removal efficiency and the adsorptive capacity, the optimum adsorbent dosage was concluded to be 1 g L^−1^.

### Effect of contact time

**Fig. 6:**
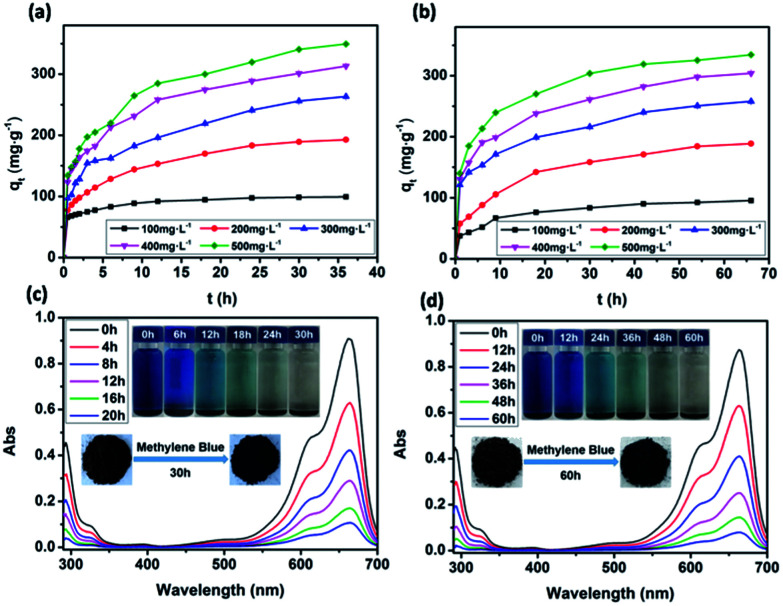
Effect of the initial concentration on MB adsorption by JLUE-COP-1 (a) and JLUE-COP-2 (b). Data are presented as mean ± SD (*n* = 3); UV-vis absorption spectra of MB adsorption by JLUE-COP-1 (c) and JLUE-COP-2 (d). Inset: photographs showing the visual color change of MB adsorption by JLUE-COPs.

The dependence of the adsorbed amount *q*_*t*_ as a function of contact time was plotted to investigate the effects of contact time on the adsorption (Fig. 6a and b). There were two phases in the adsorption progress of JLUE-COPs, including a rapid adsorption and a progressive adsorption. Due to their analogous adsorption processes, the adsorption progress of JLUE-COP-1 selected as a representative is described. In the initial adsorption of 6 h, because of the concentration difference between the adsorbent surface and aqueous solution of MB, the dyes quickly moved to the surface of the adsorbent, so that the adsorption amount rapidly increased and the MB amount rapidly decreased. Then the adsorption amount increased slowly until the adsorption equilibrium was reached. As shown in Fig. 6a, the adsorption amount stopped increasing after 24 h of contact time, indicating that equilibrium was reached. In general, the faster adsorption can be attributed to the large number of vacant surface sites; and the gradually reduced adsorption sites and repulsive forces from adsorbed dye ions result in slower adsorption. The single, smooth and continuous curve of time profile suggests that it was a uniform process and the dye on the JLUE-COP surface may be monolayer coverage.

### Effect of reagent concentrations

The effects of reagent concentration on dye adsorption were studied by keeping the concentration of dyes at 100 mg L^−1^, 200 mg L^−1^, 300 mg L^−1^, 400 mg L^−1^ and 500 mg L^−1^ at pH 8.0. From Fig. 6, it can be seen that the increased initial dye concentration induced enhanced adsorption capacity. This phenomenon can be explained by the fact that the driving force of the concentration gradient increased with the initial dye concentration. Obviously, the initial dye concentration played an important role in affecting the dye adsorption capacity and removal efficiency of JLUE-COPs (Table 1, S1 and S2†). Such high efficiencies may be attributed to the large surface area of JLUE-COPs and the high density of functional groups grafted on the JLUE-COPs. With the increase of the initial concentration of MB, the effective collision probability between the adsorbent and the dye increased, and the adsorption capacity increased. When the adsorption reached saturation, the adsorption sites of the adsorbent surface were fully occupied, meaning that there were no more vacant adsorption sites, so the adsorption quantity tended to balance. At lower dye concentrations, sufficient adsorption sites were available for adsorbing dyes. However, the numbers of dye cations were relatively higher as compared to the availability of binding sites at higher dye concentrations. Due to the limited number of binding sites present on the surface of JLUE-COPs, adsorbents become saturated at higher dye concentrations.

**Table 1:** Kinetic parameters for the adsorption of MB by JLUE-COP-1

*C*_0_ (mg L^−1^)	*q*_e,exp_ (mg g^−1^)	Pseudo-first-order kinetics			Pseudo-second-order kinetics		
		*k*_1_ (h^−1^)	*q*_e,cal_ (mg g^−1^)	*R* ^2^	*k*_2_ (g mg^−1^ h^−1^)	*q*_e,cal_ (mg g^−1^)	*R* ^2^
100	99.39	0.13	41.46	0.95	0.011	101.11	0.99
200	192.70	0.11	133.03	0.97	0.002	201.21	0.99
300	263.43	0.10	182.53	0.95	0.001	272.48	0.99
400	313.20	0.09	197.06	0.97	0.001	322.58	0.99
500	349.37	0.10	234.14	0.96	0.001	362.32	0.99

### Adsorption dynamics analysis

In order to reveal the dye adsorption behavior of JLUE-COPs, the experimental data were analyzed using two kinetic models: a pseudo-first-order kinetic model and a pseudo-second-order kinetic model. The two kinetic models could be expressed by eqn (4) and (5), respectively:^44,45^

4 ln(*q*_e_ − *q*_*t*_) = ln *q*_e_ − *k*_1_*t*

5
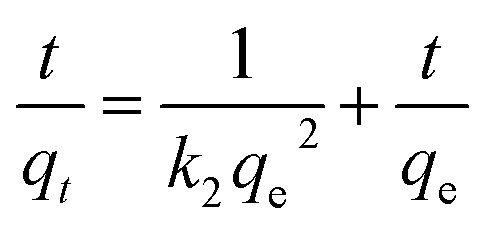
where *q*_*t*_ (mg g^−1^) and *q*_e_ (mg g^−1^) represent the same as above; *k*_1_ (h^−1^) and *k*_2_ (g mg^−1^ h^−1^) are the pseudo-first-order and the pseudo-second-order rate constants, respectively.

**Fig. 7:**
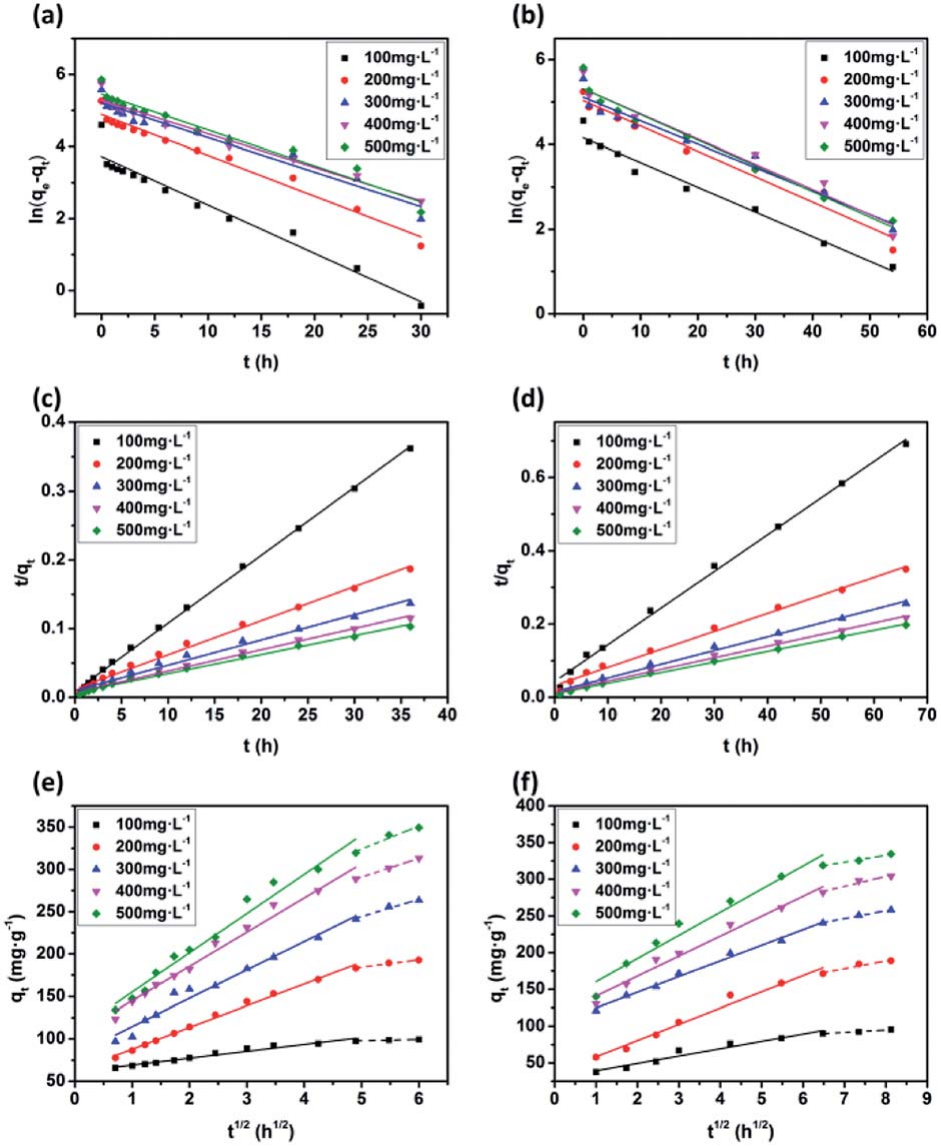
Pseudo-first-order curve-fittings for MB adsorption by JLUE-COP-1 (a) and JLUE-COP-2 (b); pseudo-second-order curve-fittings for MB adsorption by JLUE-COP-1 (c) and JLUE-COP-2 (d); intraparticle diffusion models for MB adsorption by JLUE-COP-1 (e) and JLUE-COP-2 (f) at different initial dye concentrations.

The plots of the pseudo-first-order and pseudo-second-order kinetics of MB adsorption by the two JLUE-COPs at five initial MB concentrations are shown in Fig. 7. The values of *k*_1_ and *q*_e_ for JLUE-COPs were evaluated by plotting ln(*q*_e_ − *q*_*t*_) *versus t* (Fig. 7a and b), whereas the values of *k*_2_ and *q*_e_ for JLUE-COPs were determined by plotting *t*/*q*_*t*_*versus t* (Fig. 7c and d). The results are displayed in Table 1 and S2†. For the two JLUE-COPs, the linear correlation coefficients (*R*^2^) were all in the order of the pseudo-first-order model < the pseudo-second-order model for any initial MB concentration, indicating that the dye adsorption processes in JLUE-COPs follow the pseudo-second-order kinetic model. Moreover, the calculated *q*_e_ values using the pseudo-first-order model were much lower than the experimental *q*_e_, while the calculated *q*_e_ values obtained using the pseudo-second-order model were nearly the same as the experimental values, which further proved the aforementioned conclusion. Interestingly, the kinetic data do show that the rate constant for adsorption in JLUE-COP-1 is higher than that of JLUE-COP-2; *i.e.*, the more open pores and larger specific surface area enable the fast filling of the adsorbent.

The adsorption of MB by JLUE-COPs was simulated using the intraparticle diffusion model, focusing on the diffusion mechanism. The parameters of the intraparticle diffusion model were calculated using eqn (6):^46^

6 *q*_*t*_ = *k*_*i*_*t*^1/2^ + *C*

where *k*_*i*_ (mg g^−1^ h^−1/2^) is the diffusion rate constant and *C* (mg g^−1^) is the intercept, which are proportional to the extent of boundary layer thickness.

As shown in Fig. 7e and f, the plots of *q*_*t*_ against *t*^1/2^ for each initial dye concentration exhibit a multi-linear nature. The existence of two stages can be attributed to the surface diffusion and the intraparticle diffusion, respectively. Obviously, *C* values greater than 0 suggest that the overall rate of MB uptake was controlled by both external mass transfer and intraparticle diffusion during the entire adsorption period (Table 2 and S3†). Moreover, the slopes decrease along with the increase in adsorption time, indicating decreased diffusion rates, and further acknowledging the important role of surface diffusion in the adsorption of MB by JLUE-COPs.

**Table 2:** Intraparticle diffusion model parameters for the adsorption of MB by JLUE-COP-1

*C*_0_ (mg L^−1^)	Intraparticle diffusion model					
	*k*_*i*,1_ (mg g^−1^ h^−1/2^)	*C*_1_ (mg g^−1^)	*R* ^2^	*k*_*i*,2_ (mg g^−1^ h^−1/2^)	*C*_2_ (mg g^−1^)	*R* ^2^
100	8.11	61.09	0.97	1.69	89.36	0.96
200	25.57	62.33	0.99	8.72	140.75	0.97
300	33.41	81.05	0.97	20.32	142.68	0.95
400	40.40	104.56	0.98	22.19	179.98	0.99
500	46.32	108.82	0.97	27.16	188.26	0.92

### Adsorption isotherms analysis

To further understand the adsorption of MB by the JLUE-COPs, batch experiments were performed in the temperature range of 293–323 K at different initial dye concentrations (100–400 mg L^−1^). At a constant temperature, the so-called adsorption isotherm demonstrates the relationship between the adsorption quantity and the equilibrium concentration of the solution (solid self-solution adsorption and gas–liquid interface adsorption). The adsorption isotherm is a mathematical model for describing the phase equilibrium state and the changing trend under the influence of the parameters. It represents the adsorption and separation performance of the fixed relative separation components, and provides the most basic information about thermodynamic performance. The Langmuir and Freundlich isotherm models are the most commonly used.

The Langmuir sorption isotherm is often applied for the monolayer adsorption process, while the well-known Freundlich isotherm is based on sorption on heterogeneous surface with multilayer adsorption. The Langmuir isotherm can be represented as eqn (7):^47^7
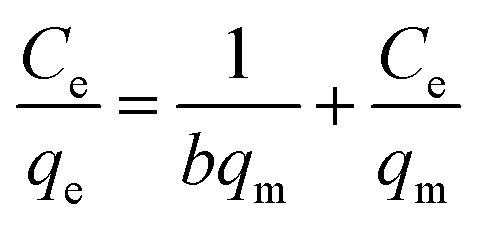
where *q*_e_ (mg L^−1^) is the adsorption capacity at equilibrium status, *C*_e_ (mg L^−1^) is the concentration of dyes in solution at equilibrium status, and *b* (L mg^−1^) is the Langmuir constant. In addition, *q*_m_ is the maximum adsorption capacity of the adsorbent.

The Freundlich isotherm model is expressed as eqn (8):^48^8
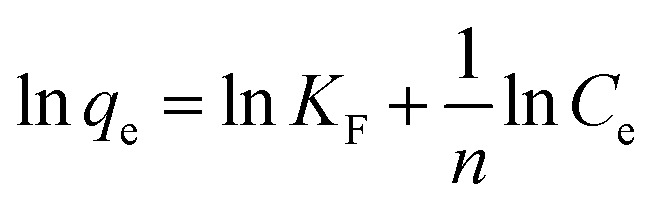
where *q*_e_ (mg g^−1^) is the adsorption capacity at equilibrium status, *C*_e_ (mg L^−1^) is the concentration of dyes in solution at equilibrium status, *K*_F_ (L g^−1^) is the Freundlich constant concerned with the adsorption capacity and 1/*n* corresponds to the heterogeneity factor.

**Fig. 8:**
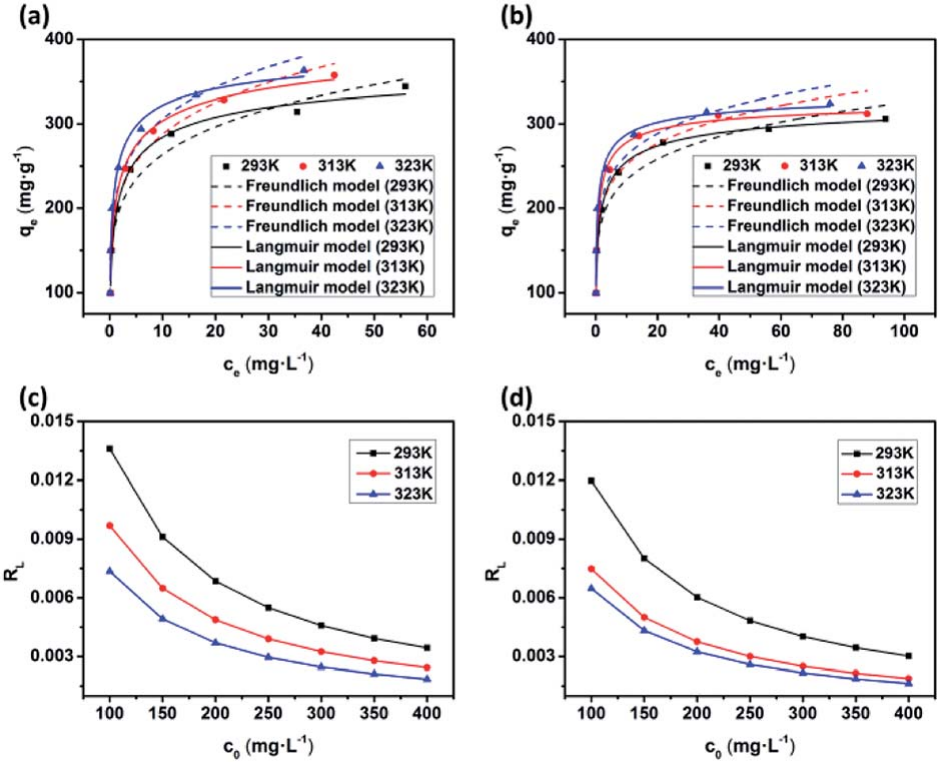
Langmuir and Freundlich adsorption isotherms of MB adsorption by JLUE-COP-1 (a) and JLUE-COP-2 (b) at different temperatures from 293 to 323 K; separation factors for MB adsorption by JLUE-COP-1 (c) and JLUE-COP-2 (d).

As shown in Fig. 8 and S11,† the adsorption performances of JLUE-COPs were interpreted using both the Langmuir and Freundlich models. Due to the lower linear correlation coefficients (*R*^2^ values) of Freundlich isotherms compared with Langmuir isotherms, Langmuir adsorption isotherms are perfect for describing MB adsorption by JLUE-COPs (Table 3 and 4 for JLUE-COP-1, Table S4 and S5† for JLUE-COP-2). In addition, it is in accordance with previous literature that monolayer adsorption often occurs in the chemical adsorption or adsorption of microporous materials. Generally, the maximum adsorption capacity *q*_m_ of MB by JLUE-COP-1 at 293 K was as high as 342.47 mg g^−1^, which is 1.11 times higher than that of JLUE-COP-2, highlighting the potential application of JLUE-COP-1 in the adsorptive removal of dyes. From the obtained values, JLUE-COPs far exceed commercial activated carbon, graphene oxide and even some MOFs (Table S7†). Similarly, the maximum adsorption capacity *q*_m_ of MB by JLUE-COP-1 was up to 359.71 and 364.86 mg g^−1^, which is 1.14 and 1.12 times higher than that of JLUE-COP-2 at 313 K and 323 K, respectively. These results are probably due to the relatively larger surface area of JLUE-COP-1 than JLUE-COP-2. In addition, from the directly proportional relationship between the maximum adsorption capacity *q*_m_ and the solution temperature, we can conclude that the affinity of the dyes for the surface of the JLUE-COPs can be improved by increasing the temperature.

**Table 3:** Adsorption parameters of Langmuir adsorption isotherm models for the adsorption of MB by JLUE-COP-1

Langmuir isotherm	Temperature (K)	*q*_m_	*K*_L_	*R* ^2^	*R*_L_
L	293	342.47	0.82	0.99	0.0034–0.0136
	313	359.71	1.02	0.99	0.0024–0.0097
	323	364.96	1.47	0.99	0.0018–0.0074

**Table 4:** Adsorption parameters of Freundlich adsorption isotherm models for the adsorption of MB by JLUE-COP-1

Freundlich isotherm	Temperature (K)	*n*	*K*_F_	*R* ^2^
F	293	5.14	169.48	0.93
	313	5.01	184.15	0.92
	323	5.17	200.73	0.90

**Fig. 9:**
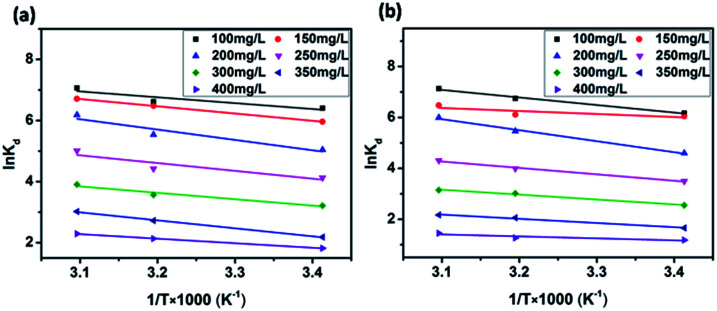
van't Hoff plots to obtain the Δ*H*^*Θ*^ and Δ*S*^*Θ*^ of MB adsorption by JLUE-COP-1 (a) and JLUE-COP-2 (b).

The affinity between MB and JLUE-COPs can be estimated with the dimensionless constant “*R*_L_”, which can be derived using the following equation:^49^9
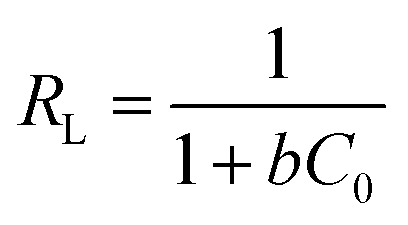
where *C*_0_ (mg L^−1^) is the initial concentration of dyes in the solution and *b* (L mg^−1^) is the Langmuir constant. The *R*_L_ values can be used to confirm whether the adsorption process is favorable or unfavorable.

As shown in Table 3 and S4,† the calculated *R*_L_ values for MB adsorption of JLUE-COP-1 and JLUE-COP-2 are in the range of 0.0018–0.0136 and 0.0016–0.0120, respectively, indicating the favorable and endothermal adsorption of dye by JLUE-COPs.

### Thermodynamic parameters

Temperature-dependent adsorption isotherms were plotted to calculate the variation in free energy (Δ*G*^*Θ*^), enthalpy (Δ*H*^*Θ*^), and entropy (Δ*S*^*Θ*^) (Fig. 9), which can be expressed as eqn (10)–(12):^50^10
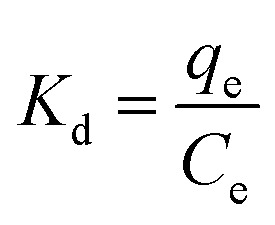

11 Δ*G*^*Θ*^ = −*RT* ln(*K*_d_)

12
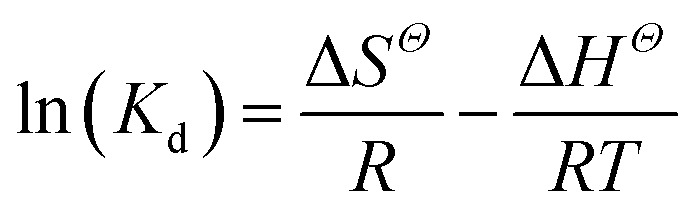
where *K*_d_ (L g^−1^) is the distribution coefficient calculated from *q*_e_/*C*_e_, *R* (8.314 J mol^−1^ K^−1^) is the gas constant and *T* (K) is the absolute temperature.

The thermodynamic parameter value changes of MB adsorption by JLUE-COPs at three adsorption temperatures are shown in Table 5 and S6†. Generally speaking, the Δ*G*^*Θ*^ value of chemisorption is greater than that of physical adsorption. The former is in the range of −400 to −80 kJ mol^−1^, while the latter is in the range of −20 to 0 kJ mol^−1^.^51^ In the present study, the Δ*G*^*Θ*^ value indicated that MB adsorption by JLUE-COPs is a physisorption process. The Δ*G*^*Θ*^ value changed with temperature a little, further confirming the physical adsorption characteristics of the adsorption process and the compensation of entropy. Positive values of Δ*H*^*Θ*^ and Δ*S*^*Θ*^ were obtained for both JLUE-COP-1 and JLUE-COP-2 at the three temperatures, implying a spontaneous endothermic reaction, that is, elevated temperatures were conducive to the adsorption process. Moreover, the magnitude of Δ*H*^*Θ*^ could also reveal the type of sorption process. The low values of Δ*H*^*Θ*^ lie within the range of 8 to 25 kJ mol^−1^, in good agreement with the physisorption results.^52^

**Table 5:** Thermodynamic parameters for the adsorption of MB by JLUE-COP-1

*C*_0_ (mg L^−1^)	Δ*G*^*Θ*^ (kJ mol^−1^)			Δ*H*^*Θ*^ (kJ mol^−1^)	Δ*S*^*Θ*^ (kJ mol^−1^ K^−1^)
	293 K	313 K	323 K		
100	−15.59	−17.22	−18.98	16.27	0.11
150	−14.52	−16.86	−18.03	19.75	0.12
200	−12.28	−14.42	−16.60	24.56	0.14
250	−10.05	−11.51	−13.46	21.70	0.11
300	−7.82	−9.28	−10.49	17.59	0.09
350	−5.32	−7.08	−8.11	21.76	0.09
400	−4.43	−5.55	−6.16	12.37	0.06

**Fig. 10:**
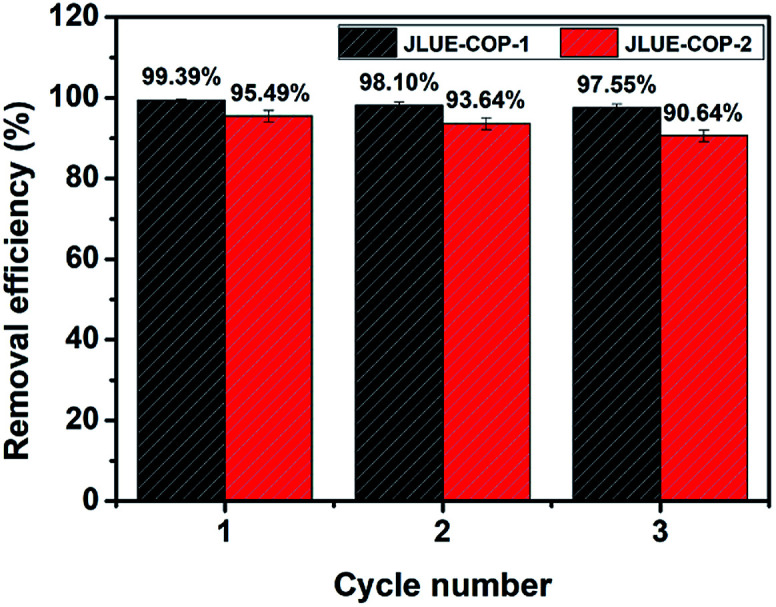
Relative adsorption capacity of JLUE-COPs after recycling. Data are presented as mean ± SD (*n* = 3).

To evaluate the practical value, the reusability of JLUE-COPs was investigated by washing the used adsorbents with acidulous methanol and drying under vacuum for the next adsorption cycle. As shown in Fig. 10, both of the adsorbed amounts of two JLUE-COPs changed little under three cycles, highlighting the promising commercial applications of JLUE-COPs. Moreover, the retained structures of the regenerated JLUE-COPs could be confirmed with the no significant spectral changes in the FT-IR spectra (data not shown).

Furthermore, adsorption experiments for cationic methylene blue (MB) dyes by JLUE-COPs in the presence of anionic methyl orange (MO) dyes were performed to investigate the selectivity of JLUE-COPs for MB dyes compared with MO dyes in solution. As shown in Fig. S12,† the adsorption capacities of JLUE-COPs for MB were much higher than those for MO. The selectivity coefficient (*S*_MB/MO_) of JLUE-COP-1 is as high as 608.44, while that of JLUE-COP-2 is as high as 92.13 (Table S8†). These results indicate the excellent selectivity of JLUE-COPs for cationic MB dyes over anionic MO dyes, demonstrating the potential application of JLUE-COPs in the separation of cationic MB dyes from anionic MO dyes.

## Conclusions

Two dual-functionalized covalent organic polymers (JLUE-COP-1 and JLUE-COP-2) were successfully prepared through heterostructural mixed linkers *via* a simple Schiff base condensation reaction. The well-designed porous COPs contain –CO–NH– and –SO_3_H groups, which strengthen the electrostatic interaction, the π–π stacking interaction and the hydrogen bonding between MB and JLUE-COPs, enhancing the adsorption properties. Meanwhile, the different configurations of the building blocks (IPA and TPA) lead to different specific surface areas and pore sizes. From analyzing the adsorption rates and maximum adsorption capacities, JLUE-COP-1 was determined to be superior to JLUE-COP-2. In addition, the thermodynamic parameters Δ*G*^*Θ*^, Δ*H*^*Θ*^ and Δ*S*^*Θ*^ indicate that the adsorption of MB by JLUE-COPs is feasible, spontaneous and endothermic. This work therefore lays the foundation for a three-component strategy to construct functionalized COPs as a new type of platform for scavenging dyes from wastewater, which can be used as a suitable alternative to commercial activated carbon.

## Conflicts of interest

There are no conflicts of interest to declare.

## Supplementary Material

RA-008-C8RA01968A-s001

## References

[cit1] Adeyemo A. A., Adeoye I. O., Bello O. S. (2012). Environ. Toxicol. Chem..

[cit2] Wang J. J., Bai R. B. (2016). Water Res..

[cit3] Gupta V. K., Suhas (2009). J. Environ. Manage..

[cit4] Haque E., Khan N. A., Park J. H., Jhung S. H. (2010). Chem.–Eur. J..

[cit5] Hasan Z., Jhung S. H. (2015). J. Hazard. Mater..

[cit6] Ghosh D., Bhattacharyya K. G. (2002). Appl. Clay Sci..

[cit7] Ai L. H., Zhang C. Y., Liao F., Wang Y., Li M., Meng L. Y., Jiang J. (2011). J. Hazard. Mater..

[cit8] Extremera R., Pavlovic I., Pérez M. R., Barriga C. (2012). Chem. Eng. J..

[cit9] Wu D. L., Zheng P. W., Chang P. R., Ma X. F. (2011). Chem. Eng. J..

[cit10] Crini G. (2006). Bioresour. Technol..

[cit11] Ma T. T., Chang P. R., Zheng P. W., Zhao F., Ma X. F. (2014). Chem. Eng. J..

[cit12] Deng H. X., Grunder S., Cordova K. E., Valente C., Furukawa H., Hmadeh M., Gandara F., Whalley A. C., Liu Z., Asahina S., Kazumori H., O'Keeffe M., Terasaki O., Stoddart J. F., Yaghi O. M. (2012). Science.

[cit13] Xiang S. C., He Y. B., Zhang Z. J., Wu H., Zhou W., Krishna R., Chen B. L. (2012). Nat. Commun..

[cit14] Zhou H. C., Long J. R., Yaghi O. M. (2012). Chem. Rev..

[cit15] Xu Y. F., Li Z., Zhang F., Zhuang X. D., Zeng Z., Wei J. J. (2016). RSC Adv..

[cit16] Zhao W. X., Hou Z. S., Yao Z. Q., Zhuang X. D., Zhang F., Feng X. L. (2015). Polym. Chem..

[cit17] He Y., Xu T., Hu J., Peng C. J., Yang Q., Wang H. L., Liu H. L. (2017). RSC Adv..

[cit18] Li X., Guo J. W., Yue H. B., Wang J. W., Topham P. D. (2017). RSC Adv..

[cit19] Eder G. M., Walkera B. R., McGrier P. L. (2017). RSC Adv..

[cit20] Zhai T. L., Du Q., Xu S., Wang Y., Zhang C. (2017). RSC Adv..

[cit21] Liu J., Chen Q., Sun Y. N., Xu M. Y., Liu W., Han B. H. (2016). RSC Adv..

[cit22] Lin S., Diercks C. S., Zhang Y. B., Kornienko N., Nichols E. M., Zhao Y., Paris A. R., Kim D., Yang P., Yaghi O. M., Chang C. J. (2015). Science.

[cit23] Chen L., Furukawa K., Gao J., Nagai A., Nakamura T., Dong Y., Jiang D. (2014). J. Am. Chem. Soc..

[cit24] Huang N., Chen X., Krishna R., Jiang D. L. (2015). Angew. Chem., Int. Ed..

[cit25] Das S., Heasman P., Ben T., Qiu S. L. (2017). Chem. Rev..

[cit26] Segura J. L., Mancheňoa M. J., Zamora F. (2016). Chem. Soc. Rev..

[cit27] Chen X., Addicoat M., Jin E. Q., Xu H., Hayashi T., Xu F., Huang N., Irle S., Jiang D. L. (2015). Sci. Rep..

[cit28] Pang Z. F., Q Xu S., Zhou T. Y., Liang R. R., Zhan T. G., Zhao X. (2016). J. Am. Chem. Soc..

[cit29] Lin L., Guan H. D., Zou D. L., Dong Z. J., Liu Z., F Xu F., Xie Z. G., Li Y. X. (2017). RSC Adv..

[cit30] Wei X., Huang T., Yang J. H., Zhang N., Wang Y., Zhou Z. W. (2017). J. Hazard. Mater..

[cit31] Yang S. T., Chen S., Chang Y. L., Cao A., Liu Y. F., Wang H. F. (2011). J. Colloid Interface Sci..

[cit32] Wei Y. B., Chen W. B., Zhao X. Y., Ding S. Y., Han S., Chen L. (2016). Polym. Chem..

[cit33] He C., Lin Z. H., He Z., Duan C. Y., Xu C. H., Wang Z. M., Yan C. H. (2008). Angew. Chem., Int. Ed..

[cit34] Leyva-Ramos R., Bernal-Jacome L. A., Acosta-Rodrigues I. (2005). Sep. Purif. Technol..

[cit35] Duan J. M., Su B. (2014). Chem. Eng. J..

[cit36] Zhang C. J., Li G. K., Zhang Z. M. (2015). J. Chromatogr. A.

[cit37] Peng Y. W., Hu Z. G., Gao Y. J., Yuan D. Q., Kang Z. X., Qian Y. H., Yan N., Zhao D. (2015). ChemSusChem.

[cit38] Wei M. M., Zhang L., Xiong Y. Q., Li J. H., Peng P. A. (2016). Microporous Mesoporous Mater..

[cit39] Hasan Z., Choi E. J., Jhung S. H. (2013). Chem. Eng. J..

[cit40] Ma J., Yang M. X., Yu F., Zheng J. (2015). Sci. Rep..

[cit41] Wang Y., Ye G. Q., Chen H. H., Hu X. Y., Niu Z., Ma S. Q. (2015). J. Mater. Chem. A.

[cit42] Crini G., Peindy H. N., Gimbert F., Robert C. (2007). Sep. Purif. Technol..

[cit43] Duan S., Li J. X., Liu X., Wang Y., Zeng S., Shao D. D., Hayat T. (2016). ACS Sustainable Chem. Eng..

[cit44] Lagergren S. (1898). K. Sven. Vetenskapsakad. Handl..

[cit45] Ho Y. S., McKay G. (1999). Process Biochem..

[cit46] Duan S. X., Tang R. F., Xue Z. C., Zhang X. X., Zhao Y. Y., Zhang W., Zhang J. H., Wang B. Q., Zeng S. Y., Sun D. Z. (2015). Colloids Surf., A.

[cit47] Langmuir I. (1918). J. Am. Chem. Soc..

[cit48] Freundlich H., Heller W. (1939). J. Am. Chem. Soc..

[cit49] Lu D. D., Cao Q. L., Cao X. J., Luo F. (2009). J. Hazard. Mater..

[cit50] Yao Y. J., Miao S. D., Yu S. M., Ma L. P., Sun H. Q., Wang S. B. (2012). J. Colloid Interface Sci..

[cit51] Kara M., Yuzer H., Sabah E., Celik M. S. (2003). Water Res..

[cit52] Yang Q. X., Zhao Q. Q., Ren S. S., Chen Z. J., Zheng H. G. (2017). Chem. Eng. J..

