# Supplementary material for: Fabrication of two dual-functionalized covalent organic polymers through heterostructural mixed linkers and their use as cationic dye adsorbents
Source: RSC Adv. 2018 May 23;8(34):19075–84. doi: 10.1039/c8ra01968a (PMC9080657; doi:10.1039/c8ra01968a)
Supplement: RA-008-C8RA01968A-s001 [file RA-008-C8RA01968A-s001.pdf]

This journal is © The Royal Society of Chemistry 2020

Supporting information for

Fabrication of two dual-functionalized covalent organic polymers through heterostructural mixed linkers and their use as cationic dye adsorbents

Jun Dong,[a] Feifan Xu,[a] Zhaojun Dong,[a] Yongsheng Zhao,[a] Yan Yan,[b] Hua Jin[a] and Yangxue Li*[a]

[a.] Key Lab of Groundwater Resources and Environment, Ministry of Education, Jilin University, 2519 Jiefangda Road, Changchun 130021, P. R. China.

[b.]State Key Laboratory of Inorganic Synthesis and Preparative Chemistry, College of Chemistry, Jilin University, Changchun 130012, P.R. China

24th January 2020

Note added after first publication: This Supplementary Information file replaces that originally published on 23rd May 2018, in which Tables S2 and S3 were incorrect.

Contents

1. PXRD spectra

2. HR-TEM images

3. EDS spectra

4. FT-IR spectra

5. TGA curves

6. Zeta potential curves

7. Chemical structures of MB and MO

8. Calibration plots of standard MB and MO

9. Adsorption isotherms analysis

10. Tables

11. Selectivity text

12. Supporting references

1. PXRD spectra

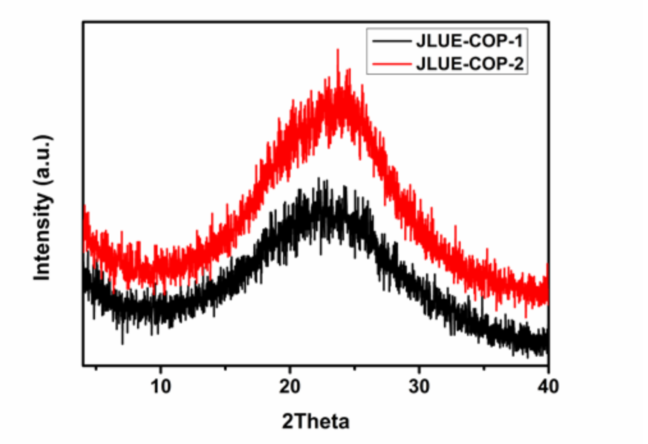

Fig. S1 PXRD spectra of JLUE-COPs.

2. HR-TEM images

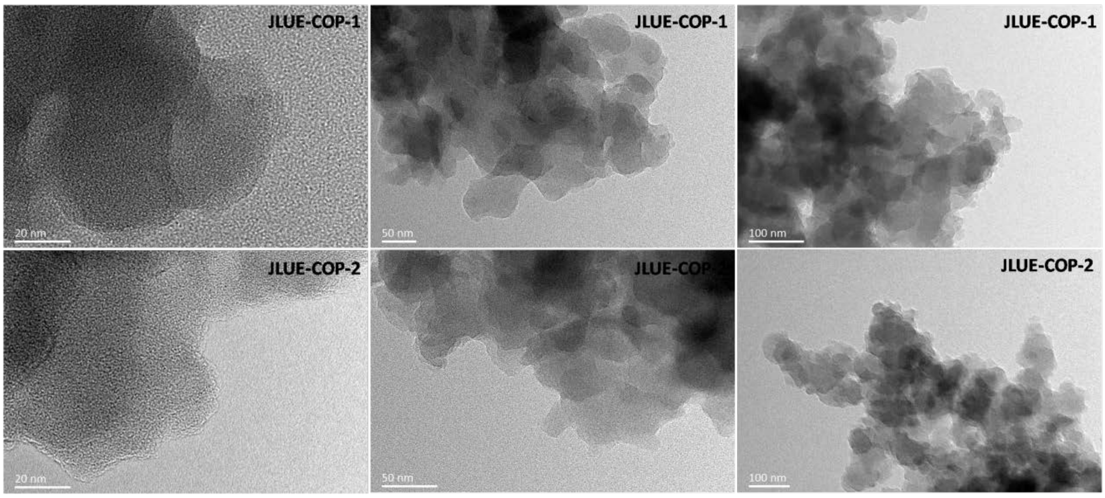

Fig. S2 HR-TEM images of JLUE-COPs.

3. EDS spectra

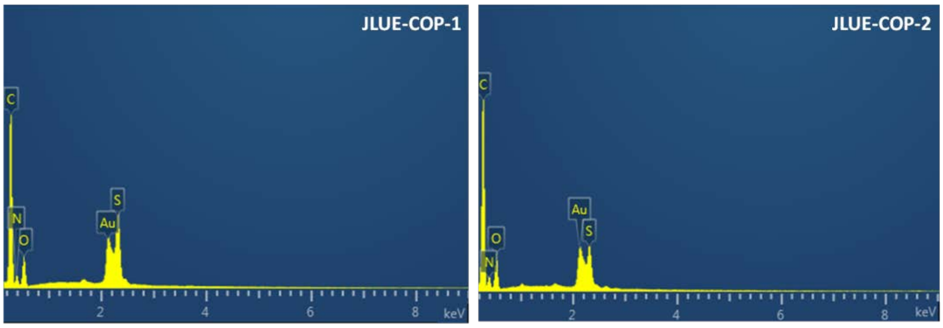

Fig. S3 EDS spectra of JLUE-COPs.

4. FT-IR spectra

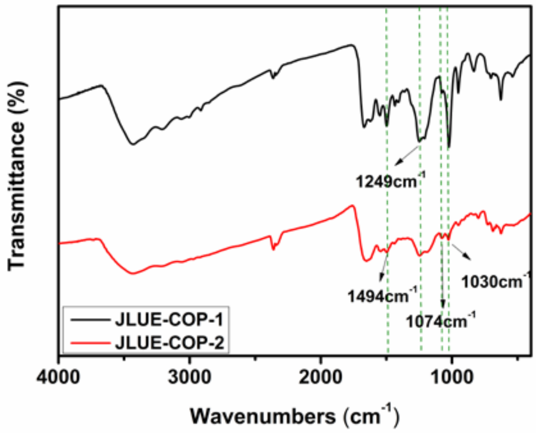

Fig. S4 FT-IR spectra of JLUE-COPs.

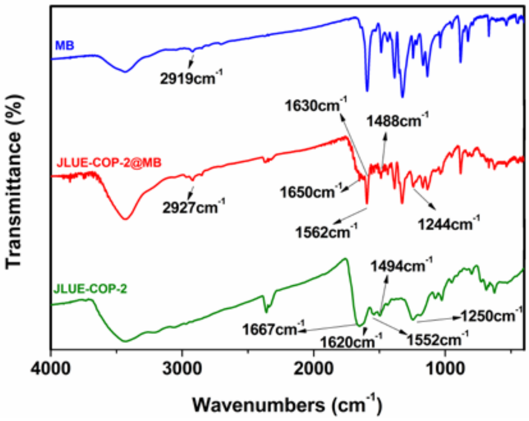

Fig. S5 FT-IR spectra of MB, JLUE-COP-2 and JLUE-COP-2@MB.

5. TGA curves

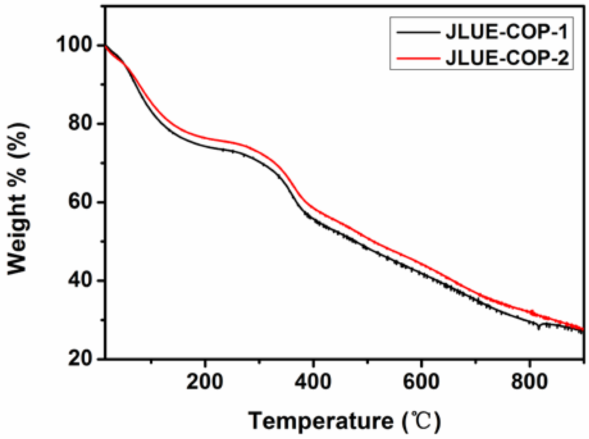

Fig. S6 TGA curve of of JLUE-COPs.

6. Zeta potential curves

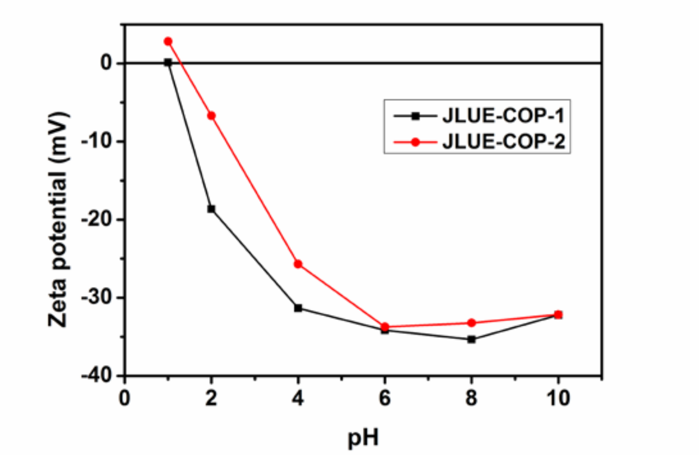

Fig. S7 Zeta potential curves versus pH of the JLUE-COPs.

7. Chemical structures of MB and MO

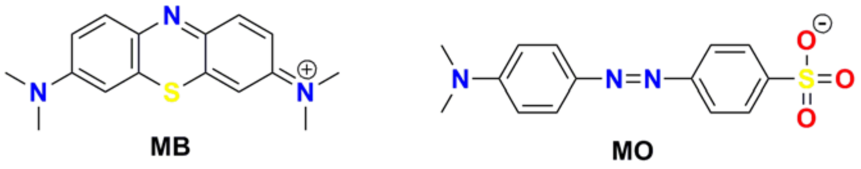

Fig. S8 Chemical structure of MB and MO.

8. Calibration plots of standard MB and MO

Calibration plot of standard MB: Five MB solutions with concentrations of 1, 2, 3, 4 and 5 mg L^{-1} at a range of pH values from 2.0 to 10.0 were prepared as standards. The calibrated plots all exhibited a good correlation coefficient (Fig. S9), which were obtained by UV-Vis spectrophotometer at 667 nm.

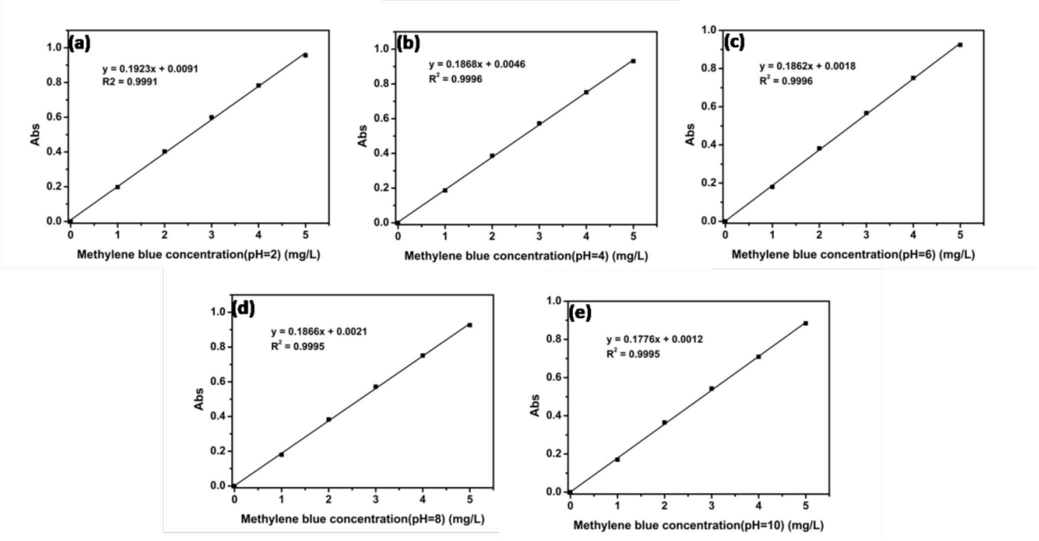

Fig. S9 Calibration plot of standard MB at pH=2 (a), pH=4 (b), pH=6 (c), pH=8 (d) and pH=10 (e), respectively.

Calibration plot of standard MO was conducted using the same method at PH=8 (Fig. S10), which were obtained by UV-Vis spectrophotometer at 465 nm.

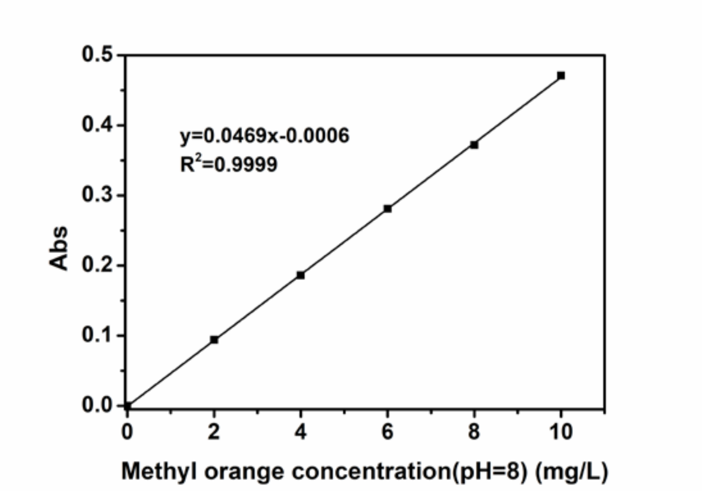

Fig. S10 Calibration plot of standard MO at pH=8.

9. Adsorption isotherms analysis

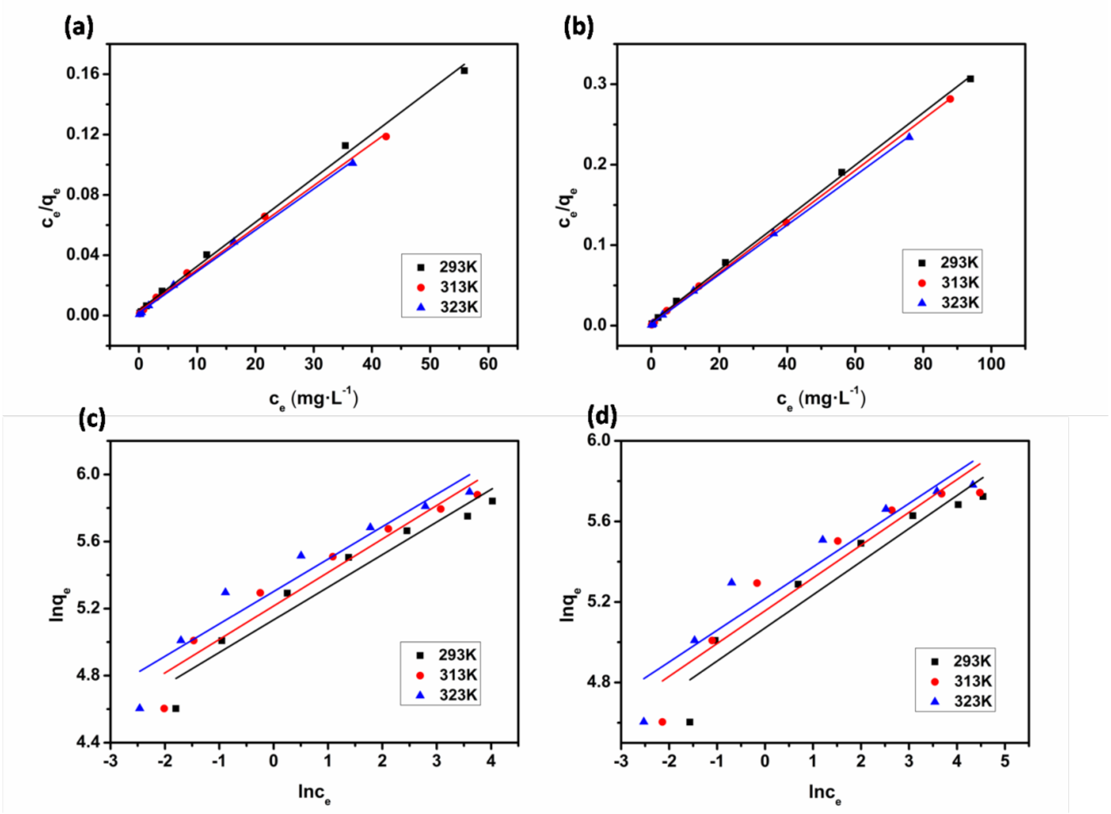

Fig. S11 Langmuir linear fittings for the MB adsorption by JLUE-COP-1 (a) and JLUE-COP-2 (b); Freundlich linear fittings for the MB adsorption by JLUE-COP-1 (c) and JLUE-COP-2 (d) at different temperatures from 293 to 323 K.

10. Tables

Table S1. Removal efficiencies of JLUE-COPs.

C_0 (mg L^{-1})	Removal Efficiency (%)	
	JLUE-COP-1	JLUE-COP-2
100	99.39	95.49
200	96.35	94.38
300	87.81	86.02
400	78.30	76.05
500	69.87	66.89

Table S2. Kinetic parameters for the adsorption of MB by JLUE-COP-2.

C_0 (mg·L^{-1})	$q_{e,exp}$ (mg·g^{-1})	Pseudo-first-order kinetics			Pseudo-second-order kinetics		
		k_1 (h^{-1})	$q_{e,cal}$ (mg·g^{-1})	R^2	k_2 (g·mg^{-1}·h^{-1})	$q_{e,cal}$ (mg·g^{-1})	R^2
100	95.49	0.058	63.89	0.97	0.0023	100.00	0.99
200	188.76	0.060	153.72	0.97	0.0007	203.67	0.99
300	258.05	0.055	165.95	0.95	0.0009	267.38	0.99
400	304.20	0.059	203.21	0.95	0.0007	316.46	0.99
500	334.44	0.060	202.99	0.96	0.0008	346.02	0.99

Table S3. Intraparticle diffusion model parameters for the adsorption of MB by JLUE-COP-2.

C_0 (mg·L^{-1})	Intraparticle diffusion model					
	$k_{i,1}$ (mg·g^{-1}·$h^{-1/2}$)	C_1 (mg·g^{-1})	R^2	$k_{i,2}$ (mg·g^{-1}·$h^{-1/2}$)	C_2 (mg·g^{-1})	R^2
100	9.98	29.43	0.95	3.20	69.28	0.97
200	21.30	36.31	0.97	9.35	101.97	0.90
300	22.04	103.40	0.99	10.78	170.84	0.99
400	27.21	113.48	0.97	10.85	195.21	0.91
500	31.56	128.73	0.95	13.60	257.85	0.97

Table S4. Adsorption parameters of Langmuir adsorption isotherm models for the adsorption of MB by JLUE-COP-2.

Langmuir isotherm	Temperature (K)	Q_m	K_L	R^2	R_L
L	293	306.75	0.79	0.99	0.0030～0.0120
	313	314.47	1.41	0.99	0.0019～0.0075
	323	325.73	1.39	0.99	0.0016～0.0065

Table S5. Adsorption parameters of Freundlich adsorption isotherm models for the adsorption of MB by JLUE-COP-2.

Freundlich isotherm	Temperature (K)	n	K_F	R^2
F	293	6.08	159.36	0.90
	313	6.14	173.57	0.89
	323	6.35	184.34	0.88

Table S6. Thermodynamic parameters for the adsorption of MB by JLUE-COP-2.

C_0 (mg L^{-1})	ΔG^{Θ} (KJ • mol^{-1})			ΔH^{Θ} (KJ • mol^{-1})	ΔS^{Θ} (KJ • mol^{-1} • K^{-1})
	293K	313K	323K		
100	-15.03	-17.56	-19.15	24.78	0.14
150	-14.74	-15.90	-17.40	10.14	0.08
200	-11.20	-14.21	-16.09	25.15	0.16
250	-8.51	-10.38	-11.57	21.04	0.10
300	-6.20	-7.84	-8.44	15.94	0.08
350	-4.04	-5.35	-5.82	13.60	0.06
400	-2.88	-3.30	-3.90	6.59	0.03

Table S7. Adsorption capacity q_m of various absorbents of MB.

S.N.	Adsorbents	Adsorption capacity (mg g^{-1})	Reference
1	GO-PVA	178.5	1
2	GO	250	2
3	Fly ash A	6.0.	3
4	Zeolite NaA	64.8	4
5	Commercial activated carbon	22.3	5
6	M-MWCNTs	48.06	6
7	Diatomite	156.6	7
8	Tea waste	85.5	8
9	Iron terephthalate (MOF-235)	187	9
10	ZIF-8	19.5	10
11	ZIF-8 derived carbon	186.3	10
**12**	**JLUE-COP-2**	**306.75**	**This work**
**13**	**JLUE-COP-1**	**342.47**	**This work**

11. Selectivity text

The selectivity text of MB adsorption from aqueous solution containing MO was carried out at pH=8. The initial concentration of MB and MO both are 100 mg L^{-1}, and the residual concentration in the supernatant of MB and MO was determined by UV-vis spectrophotometer at 667 nm and 465 nm , respectively.

The selectivity coefficient ($S_{MB/MO}$) for MB relative to MO is defined as:

$$S_{MB/MO} = \frac{K_d^{MB}}{K_d^{MO}} \tag{1}$$

$$K_d = \frac{(C_0 - C_e)V}{C_e m} \tag{2}$$

where K_d^{MB} and K_d^{MO} are the distribution ratio of MB and MO in adsorbent and solution, respectively.

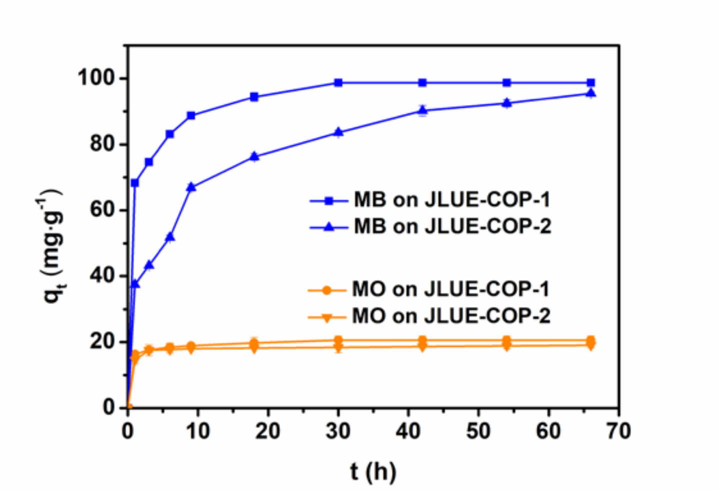

Fig. S12 Effect of the contact time on the adsorption of MB and MO by JLUE-COPs. Data are presented as mean ± SD (n = 3).

Table S8. The selectivity coefficients of MB for MO by JLUE-COPs.

	JLUE-COP-1		JLUE-COP-2	
	MB	MO	MB	MO
q_e(mg g^{-1})	99.39	21.04	95.49	19.06
K_d	164.28	0.27	21.19	0.23
$S_{MB/MO}$	608.44		92.13	

12. Supporting references

1. J. Dai, T. Huang, S. Q. Tian, Y. J. Xiao, J. H. Yang, N. Zhang and Z. W. Zhou, *Mater. Des.*, 2016, **107**, 187–197.

2. Q. L. Fang and B. L. Chen, *J. Mater. Chem. A*, 2014, **2**, 8941–8951.

3. P. Janos, H. Buchtová and M. Rýznarová, *Water Res.*, 2003, **37**, 4938–4944.

4. Z. Zhang and J. Kong, *J. Hazard. Mater.*, 2011,**193**, 325–329.

5. N. Sapawe, A. A. Jalil, S. Triwahyono, M. I. A. Shah, R. Jusoh, N. F. M. Salleh, B. H. Hameed and A. H. Karim, *Chem. Eng. J.*, 2013, **229**, 388–398.

6. L. H. Ai, C. Y. Zhang, F. Liao, Y. Wang, M. Li, L. Meng and J. Jiang， *J. Hazard. Mater.*, 2011, **198**, 282–290.

7. M. A. Al-Ghouti, M. A. M. Khraisheh, S. J. Allen and M. N. Ahmad, *J. Environ. Manage.*, 2003, **69**, 229–238.

8. M. T. Uddin, M. A. Islam, S. Mahmud and M. Rukanuzzaman, *J. Hazard. Mater.*, 2009, **164**, 53–60.
9. E. Haque, J. W. Jun and S. H. Jhung, *J. Hazard. Mater.*, 2011, **185**, 507–511.
10. Z. Abbasi, E. Shamsaei, S. K. Leong, B. Ladewig，X. W. Zhang and H. T. Wang，*Microporous Mesoporous Mater.*, 2016, **236**, 28–37.
